# Managing urban runoff in residential neighborhoods: Nitrogen and phosphorus in lawn irrigation driven runoff

**DOI:** 10.1371/journal.pone.0179151

**Published:** 2017-06-12

**Authors:** Gurpal S. Toor, Marti L. Occhipinti, Yun-Ya Yang, Tammy Majcherek, Darren Haver, Lorence Oki

**Affiliations:** 1 Soil and Water Quality Laboratory, Gulf Coast Research and Education Center, University of Florida, Institute of Food and Agricultural Sciences, Wimauma, FL, United States of America; 2 South Coast Research & Extension Center, University of California Cooperative Extension, 7601 Irvine Blvd, Irvine, CA, United States of America; 3 Department of Plant Sciences, University of California, Davis, Davis, CA, United States of America; The Education University of Hong Kong, HONG KONG

## Abstract

Sources and mechanisms of nutrient transport in lawn irrigation driven surface runoff are largely unknown. We investigated the transport of nitrogen (N) and phosphorus (P) in lawn irrigation driven surface runoff from a residential neighborhood (28 ha) of 56% impervious and 44% pervious areas. Pervious areas encompassing turfgrass (lawns) in the neighborhood were irrigated with the reclaimed water in common areas during the evening to late night and with the municipal water in homeowner’s lawns during the morning. The stormwater outlet pipe draining the residential neighborhood was instrumented with a flow meter and Hach autosampler. Water samples were collected every 1-h and triple composite samples were obtained at 3-h intervals during an intensive sampling period of 1-week. Mean concentrations, over 56 sampling events, of total N (TN) and total P (TP) in surface runoff at the outlet pipe were 10.9±6.34 and 1.3±1.03 mg L^–1^, respectively. Of TN, the proportion of nitrate–N was 58% and other–N was 42%, whereas of TP, orthophosphate–P was 75% and other–P was 25%. Flow and nutrient (N and P) concentrations were lowest from 6:00 a.m. to noon, which corresponded with the use of municipal water and highest from 6:00 p.m. to midnight, which corresponded with the use of reclaimed water. This data suggests that N and P originating in lawn irrigation driven surface runoff from residential catchments is an important contributor of nutrients in surface waters.

## Introduction

Surface waters increasingly receive nutrients from terrestrial environments resulting in deterioration of water quality [1–3]. Water running over the land surface carries nutrients such as nitrogen (N) and phosphorus (P) to adjacent water bodies causing harm to aquatic ecosystems [4–7]. Urban stormwater runoff studies in the U.S. have proven to be too broad to predict nutrient concentrations in local water bodies, although they have brought attention to the point and nonpoint source pollution impacts on receiving waters [8, 9]. Regional variability in nutrient concentrations in water bodies due to the influence of climate, soils, and anthropogenic activities (e.g., irrigation and fertilizer application) has illustrated the need for local studies to accurately examine nutrient transport in stormwater runoff in the urban drainage networks [3].

Understanding the sources and transport pathways of nutrients can help to develop and fine-tune practices to protect sensitive water bodies. In urban systems, natural hydrology is altered by impervious areas (e.g., pavement and rooftops), which decreases infiltration, increases runoff, and shortens the residence time of nutrients in the soil and runoff waters [10, 11]. Increase in impervious surfaces in urban watersheds has been related to increased concentrations of N and P in surface runoff [12–14] and a decline in biodiversity in streams [15]. To date, most water quality studies have investigated runoff from broad land uses such as forest, agricultural, and urban. This approach assumes that the developed or urban land use is spatially homogeneous within the watershed. Perhaps, a better approach may be to characterize specific land uses, such as residential neighborhoods, in the urban drainage network.

In the urban landscapes, pollutants of concern include nutrients, bacteria, organic compounds, heavy metals, and sediments [16–18]. In residential catchments, the main sources of nutrients are lawn fertilizer, vehicular emissions, atmospheric deposition, organic matter (i.e. lawn clippings, tree leaves), recycled or reclaimed water used for irrigation, and pet waste [19–26].

The irrigation of urban landscapes with treated wastewater (hereafter referred to as reclaimed water) is an alternative method for relieving water shortages in the arid and semiarid areas of California and other regions [27]. Nutrients (N and P) in reclaimed water can help meet partial nutrition needs of urban lawns, however, there are the risks of nutrient imbalances and runoff losses when using reclaimed water [28]. Nitrogen is generally a limiting nutrient in marine ecosystems and P a limiting nutrient in freshwater ecosystems, whereas estuarine ecosystems can show both N and P limitation at varying spatio-temporal scales [21, 29–30]. When managing N and P in surface waters, Conley et al. [1] suggests a dual approach which considers both N and P and seasonal shifts in limiting nutrients as these may help to reduce impacts on water quality.

Our objective in this study was to investigate the transport of N and P forms in runoff driven by lawn irrigation in a residential catchment. Our hypothesis was that intensive sampling (at 3-h intervals) in the residential catchment can help distinguish the patterns of N and P forms in runoff that are not detected with more conventional infrequent coarse sampling such as weekly, bi-weekly, or monthly. To our knowledge, this study is the first report of N and P forms in surface runoff driven by lawn irrigation with a mix of reclaimed water and municipal water at a residential catchment scale.

## Materials and methods

### Site description

**Fig 1:**
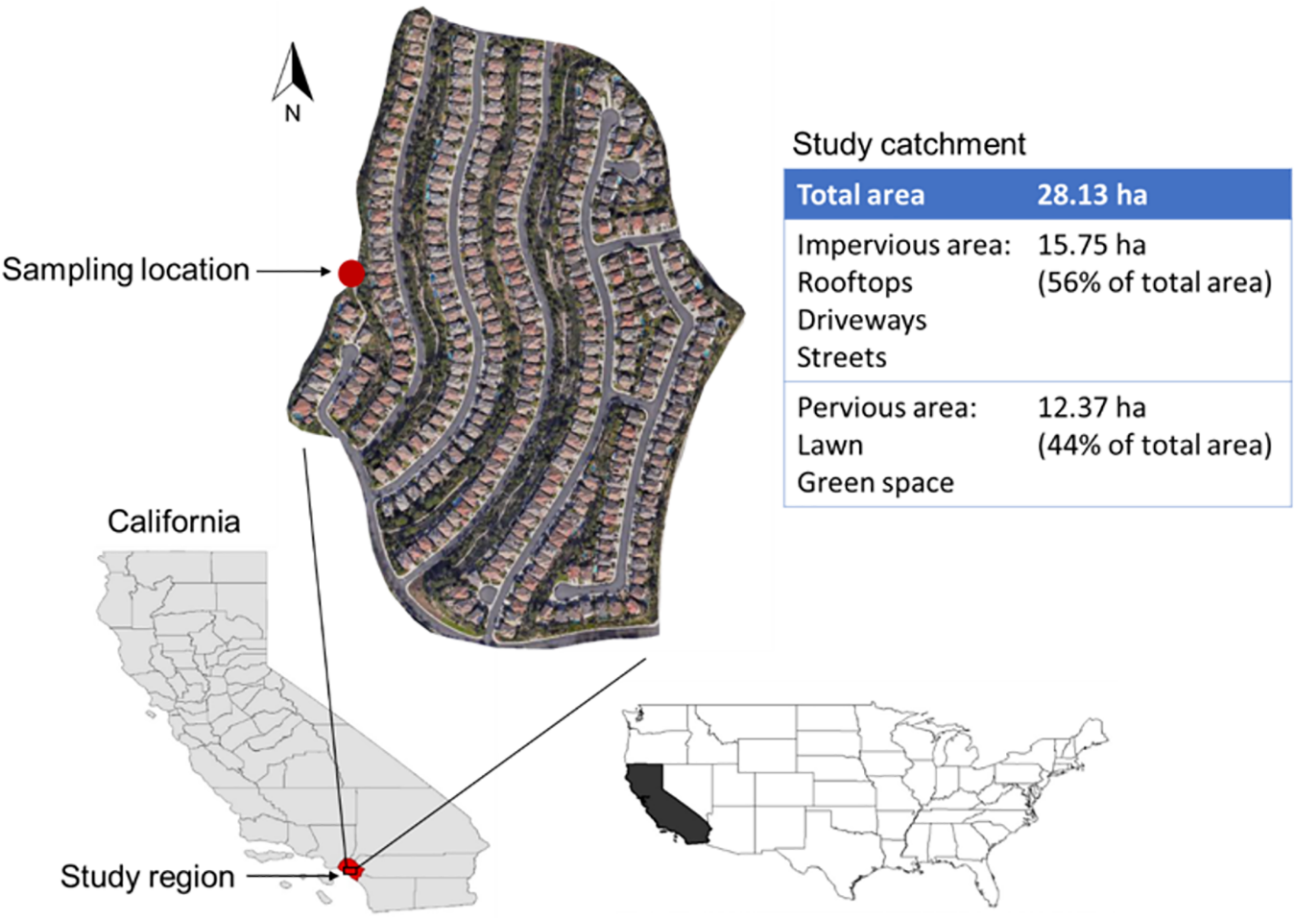
The residential study area within the Aliso Creek Watershed in southern California where intensive water quality sampling was conducted in June 2008.

The study site is located in a coastal residential community that drains into the Aliso Creek watershed in Orange County, California, United States (Fig 1), which is 80 km south of Los Angeles and 105 km north of San Diego. The study was conducted on a private property managed by the homeowners association (HOA) who gave the necessary permission to conduct water sampling in the residential catchment. In-stream sampling conducted in the watershed downstream of the study site indicated that the main contaminants of concern are N, P, bacteria, and selenium [31]. The residential catchment native soils are typically dominated with clay (20%–45%) and low organic matter (less than 2%) and have moderate to low permeability that are correlated with high runoff potential. The residential neighborhood is located on steep and rocky terrain with slopes ranging from 15 to 75% [32]. In urban landscapes, native soils are considerably altered prior to landscaping for improving the foundation, which often result in greater compaction, reduction in infiltration, and increase in surface runoff. After establishment, individual homeowners amend turf and landscape plants with fertilizers, organic materials (composts) at different frequencies and rates likely resulting in considerable variability in physical, chemical, and microbiological properties among parcel soils.

The study area has a semi-arid Mediterranean climate with warm dry summers and cool wet winters. Due to the dry summers, landscapes in California require supplemental irrigation to maintain plant health and aesthetic appearance. Historic 40-year rainfall data from a local gauging station showed annual average precipitation of approximately 38 cm [33]; of which 36 cm (95%) occurred during the wet season (October–April) and 2 cm (5%) occurred during the dry season (May–September). Mean annual air temperature was 20°C during the dry season and 15°C during the wet season.

The study site (total area: 28.13 ha) is a residential HOA community consisting of 307 single-family homes constructed in the mid to late 1990’s and does not include other land use types [34]. The HOA in the United States is defined as an organization in a residential community consisting of single-homes family or multiple units, which makes and enforces rules for the properties (i.e. homes) within the subdivision. Median household income in the study area was $95,498 in 2008 [35]. The parcels within the study were largely owner occupied and had similar, mature, and well-maintained yards. Commercial multispectral aerial imagery from QuickBird (QB) and geographic information system (GIS) raster analysis determined that single-family home sites (including homes, private yards, and driveways) occupied 53% of the total area, streets occupied 22%, and the remaining 25% was classified as “other” consisting mainly of common lawn and green space. Impervious surface area in the entire neighborhood including rooftops, driveways, and streets was estimated to be 56%, with the remainder 44% as pervious areas.

Residents in the neighborhood used municipal (potable) water for landscape irrigation, whereas the common areas managed by HOA were irrigated with reclaimed water that originated from a wastewater treatment plant operated by the South Orange County Wastewater Authority (SOCWA). The utility provided basic information on the nutrient content of the reclaimed water, with the average TN of approximately 38 mg/L and the average TP of 6 mg/L at the treatment plant (personal communication, SOCWA). According to data provided by the local water district, the average monthly potable water use per household at the time of the study (summer, May to September 2008) was 56,070 L and ranged from 48,100 L to 66,800 L [36]. The access to detailed reclaimed water use data in the catchment was not available. To reduce the occurrence of human contact with contaminants present in the reclaimed water, the SOCWA prohibits the use of reclaimed water for lawn irrigation between the hours of 9:00 a.m. and 6:00 p.m. The exact irrigation schedules could not be determined for the site, although it was observed that irrigation of common areas with reclaimed water occurred from 10:00 p.m. to 5:00 a.m, whereas most of the residential irrigation with potable water occurred from 5:00 a.m. to 9:00 a.m.

### Flow measurement

The continuous flow at the outflow pipe of the residential catchment was due to the lawn irrigation as no rainfall occurred during the study period. In this catchment, sewer and stormwater pipes are separate, as this is also norm for most of California. Runoff from the residential catchment flows in a gutter and storm water system, which was outfitted with a 107-cm storm outflow pipe that discharged water into the drainage network of Aliso Creek Watershed. Flow was measured continuously at 2-min intervals from October 2007 to September 2008 at the outflow pipe with an automated in-situ area-velocity sensor placed in the bottom of the pipe (Hach Sigma 950 Flowmeter, Hach Company, Loveland, CO). The flow (*Q*) was determined by multiplying the mean water velocity (*V*) and area (*A*) of the outfall pipe [37]. The flow meter was checked at weekly intervals and data were downloaded. Due to the large volume of flow data collected, 2-min measured flow data were aggregated to hourly flows. Recorded negative flows were considered erroneous and omitted. Using the flow depth and the size, shape, slope, and roughness of the channel, the Manning formula was used as a substitute of confirming unreliable field flow data [37]. It was determined that positive measured flows based on area-velocity at the outflow pipe were usable when compared to calculated flows using the measured water depth and pipe properties in the Manning formula [38] (data not shown). During a separate concurrent study, water was captured during low flow periods to measure flow to further validate the accuracy of the calculated flow of the flow meter. See Supplementary information section S1 File for details.

### Sample collection and processing

Intensive sampling was conducted at 3-h intervals starting at 9:00 a.m. on June 16, 2008 and ending at 9:00 a.m. on June 23, 2008. As the study area only receives 38 cm of annual rainfall, of which, only 2 cm occurs during the dry season of May to September [33], we can expect that runoff is similar in nature during this dry period of the year. Therefore, the selected time period is appropriate to determine the nature of runoff in this residential neighborhood. The autosampler (Hach Sigma 900Max Sampler, Hach Company, Loveland, CO) was calibrated to take a 300 mL sample every 1-h and then create a triple composite sample of 900 mL every 3-h. Samples were removed from the autosampler every 24-h. The core of the sampling machine was iced and new ice was added every 24-h to maintain the integrity of the samples. The pick up tube from the autosampler was placed in the outfall pipe to collect runoff waters before the runoff entered Wood Creek, a tributary to Aliso Creek.

After collection, samples were transferred on ice to the laboratory where Environmental Protection Agency (EPA) standard analyses protocols [39] were used to analyze total N (40 CFR 141), nitrate–N (NO_3_–N) (EPA 350.1), total P (EPA 365.1), orthophosphate–P (PO_4_–P) (EPA 365.3), total suspended solids (TSS) (EPA 160.2), turbidity (EPA 180.1), electrical conductivity (EC), and total organic carbon (TOC) (EPA 9060A). Other–N (organic and ammonium) was calculated as the difference between TN and NO_3_–N. Other–P (particulate and organic) was calculated as the difference between TP and PO_4_–P.

### Statistical analysis

The one-way Analysis of Variance (ANOVA) Tukey-Kramer HSD (honest significant difference) test was used to examine the significant differences (*p* < 0.05) of measured variables during the sampling time. Measured data met the assumption of normality for parametric statistical analyses. All statistical analyses were performed using the JMP statistical software package (JMP Pro 12, SAS Institute).

## Results and discussion

### Flow dynamics in the residential neighborhood

**Fig 2:**
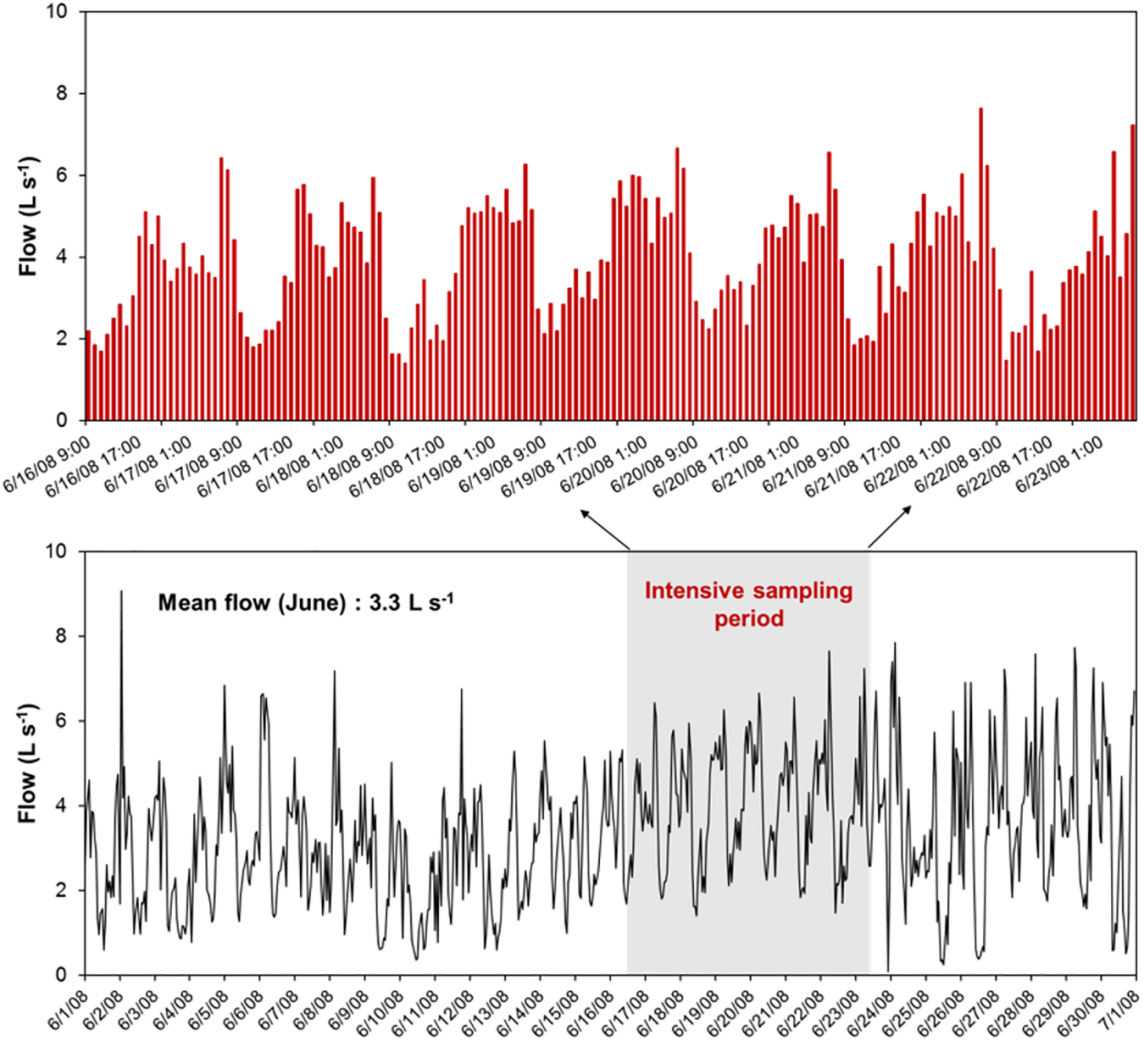
Measured flow at 1-hour intervals at the outflow pipe draining a southern California residential neighborhood during June 2008 (bottom graph) with highlighted intensive sampling period of 1-week from June 16 to 23 2008 (top graph).

The range of flow observed from October 2007 to September 2008 varied from 0 to 364.57 L s^–1^. In general, the mean monthly flow was lower in October 2007 (1.93 L s^–1^) and September 2008 (1.99 L s^–1^), whereas it was higher in January 2008 (8.18 L s^–1^). The mean monthly flow during June 2008 (3.3 L s^–1^) compared well with the mean annual flow (3.53 L s^–1^) and the variability in flow was similar to the variability observed during the dry season (S1 Table). During the sampling period, flow varied from 1.55 to 7.23 L s^–1^ and showed a diurnal increase and decrease, with the lowest flow measured each day just before noon (Fig 2). After 12:00 p.m., flow continued to increase and the maximum flow occurred each morning near 6:00 a.m. The diurnal cycle was consistent throughout the intensive sampling period as well during the entire month of June. This flow pattern showed that irrigation schedules dictated the daily flow pattern. For example, the common areas were irrigated with reclaimed water from 10:00 p.m. to 5:00 a.m. and homeowner lawns were irrigated with potable water from 5:00 a.m. to 9:00 a.m. The highest flow peak at 6 a.m. was attributed to the runoff caused by overlap of irrigation with reclaimed and portable water. This variation in daily flow is not uncommon in urban studies. For example, a study in Los Angeles, California reported that dry weather flows in arid urban watersheds can change by as much as 40% during the course of a single day [40]. Using the monthly mean flows measured in this residential catchment from October 2007 to September 2008, it was estimated that dry season (May–September) contributed 37% and the wet season (October–April) contributed 63% of annual flow (S1 Table). Our dry flow values are within 10 to 50% of annual dry season flow range reported by McPherson et al. [41]. The detailed flow, pH, EC, TOC, and TSS data collected at 3-h intervals over the study period is reported in S2 Table.

### Nitrogen concentrations and loading in residential runoff

The mean (n = 56) concentrations of TN, NO_3_–N, and other–N (organic and ammonium) at the outlet pipe in the residential catchment during the intensive sampling period were 10.85, 5.42, and 5.43 mg L^–1^, respectively (Table 1). The NO_3_–N:TN was 0.58 and other–N:TN was 0.42. The lowest concentrations of TN, NO_3_–N, and other–N were 4.27, 2.42, and 0.09 mg L^–1^, respectively.

**Table 1:** Concentrations of nitrogen, phosphorus, and total suspended solids at the outflow pipe draining a southern California residential neighborhood during one-week of intensive sampling in June 2008.

Nutrient Concentration	Total N	Nitrate–N	Other –N	Total P	Orthophosphate–P	Other –P	Total suspended solids
	mg L^–1^						
Mean	10.85	5.42 (0.58)^a^	5.43 (0.42)	1.27	0.82 (0.75)	0.45 (0.25)	52.18
Minimum	4.27	2.42	0.09	0.51	0.41	0.02	3.44
Maximum	29.80	9.50	21.82	7.47	1.79	7.06	274.36
Standard deviation	6.34	1.72	5.24	1.03	0.36	1.00	59.11

^a^ Data in parentheses are ratios of nitrate–N:TN, other–N:TN, orthophosphate–P:TP, and other–P:TP during the study period.

**Fig 3:**
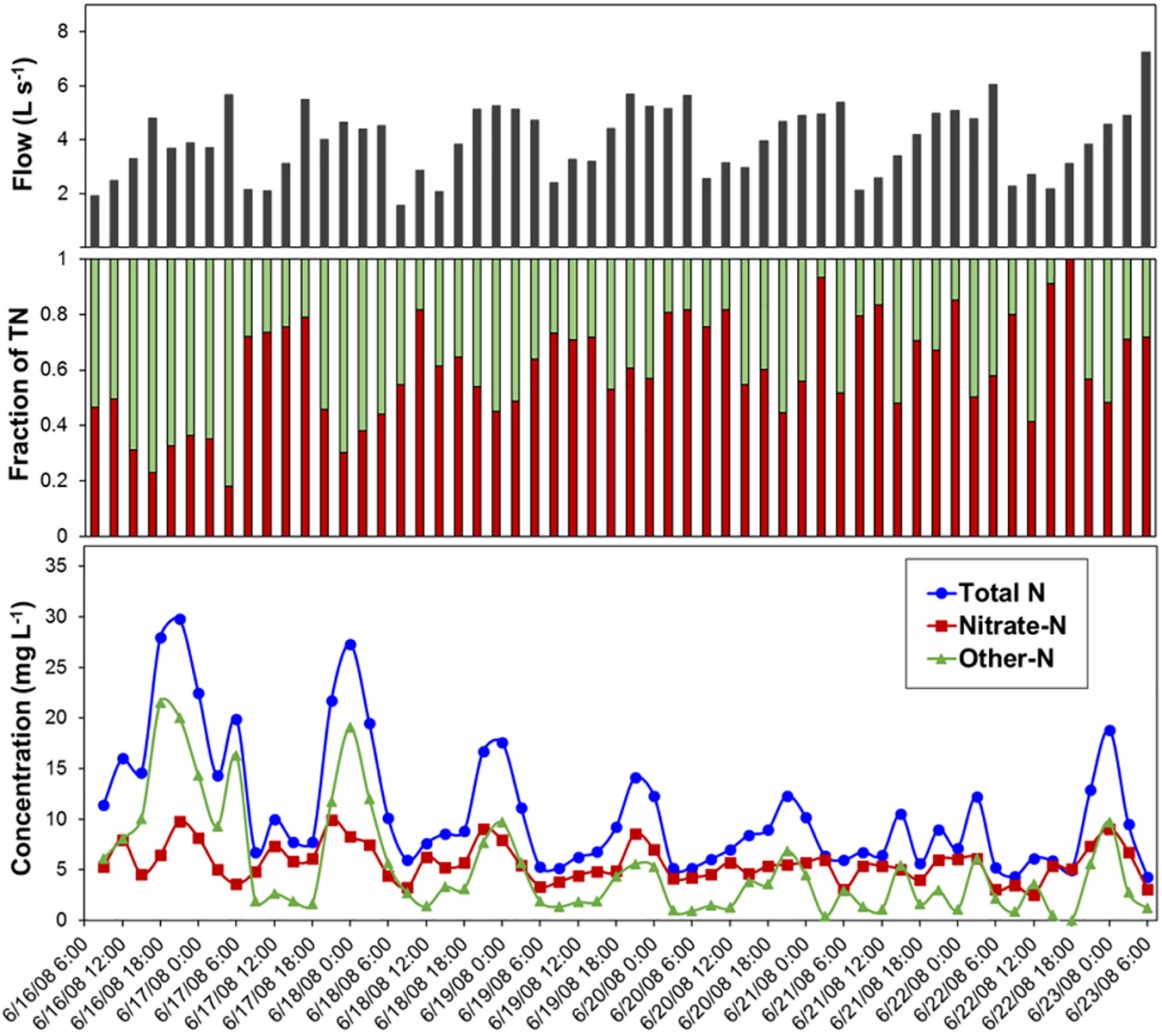
From top to bottom represents measured flow, fraction of NO_3_–N (red) and other–N (green), and concentrations of N forms in residential runoff collected at 3-hour intervals over 56 sampling events from outflow pipe draining a residential catchment during an intensive sampling period of 1-week (June 16 to 23 2008).

**Fig 4:**
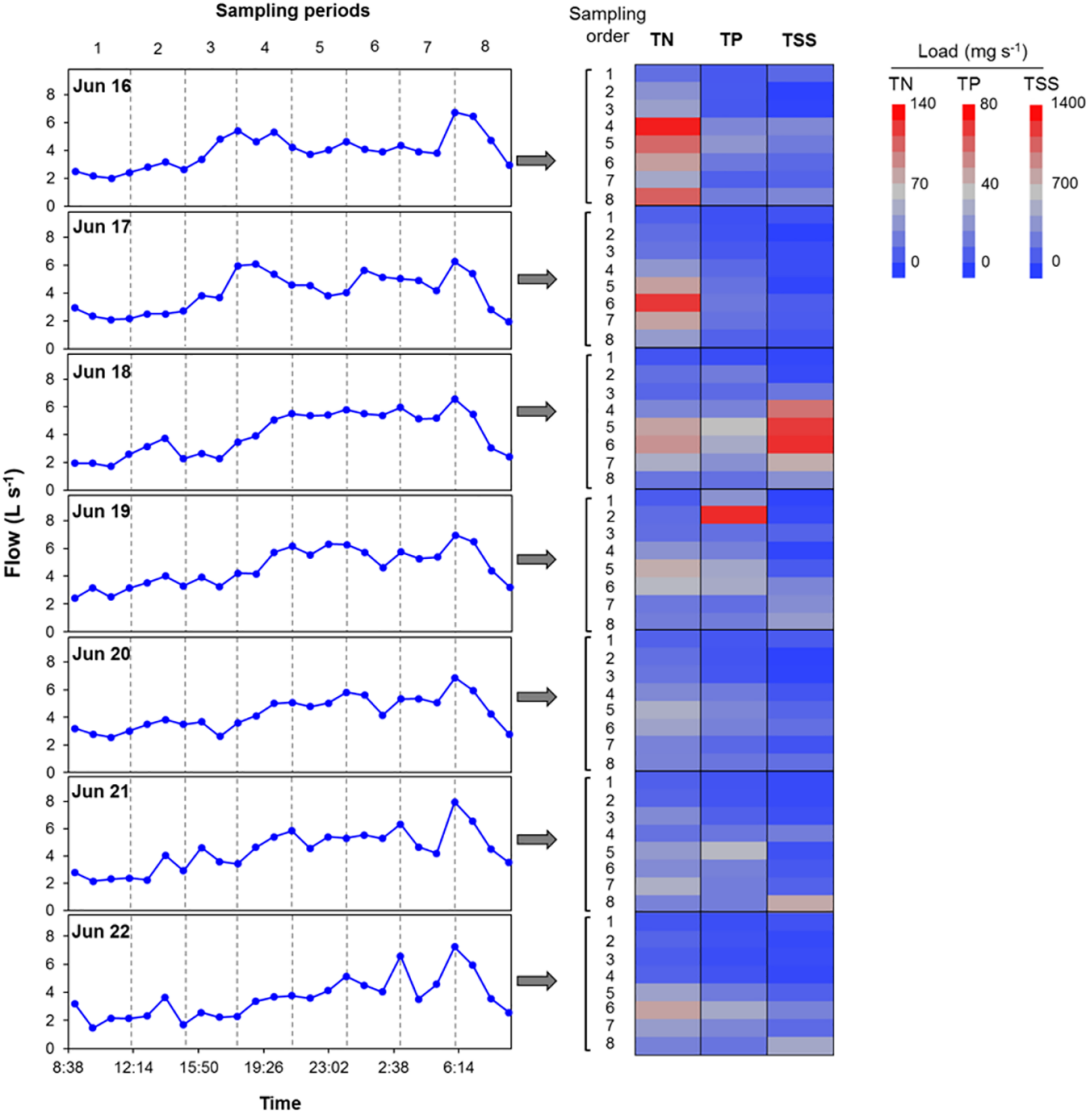
Measured flow at 1-hr intervals and distribution of loads of TN, TP, and TSS in residential runoff collected at 3-hour intervals over 56 sampling events from outflow pipe draining a residential catchment during an intensive sampling period of 1-week (June 16 to 23 2008).

Nitrogen concentrations exhibited a diurnal pattern during each 24-h period with highest recorded values between 8:00 p.m. and midnight and lowest values between 6:00 a.m. and noon (Fig 3). Concentrations of N were greater in the beginning (June 16) than near the end of the sampling period (June 23). Concentration of TN and other–N were significantly (*p* <0.05) greater in day 1 than remainder of the days (Fig 3 and S1 Fig). Nitrogen loading exhibited daily variations that were similar to flow and N concentrations. The mean loading of TN was 3.84 kg day^–1^ (S3 Table). Lowest TN loads occurred from 6:00 a.m. to noon and highest loads frequently occurred from 6:00 p.m. to midnight each day (Fig 4). Total N loads increased in response to sampling events on June 18 to June 20, 2008. The detailed concentrations and proportions data of N forms collected at 3-h intervals over the study period is reported in S4 Table.

Of 56 sampling events, nearly 80% of samples had NO_3_–N:TN of 0.50 or more, whereas the remainder of samples had other–N as the dominant form (Fig 3). Twelve of these samples coincided with the higher TN concentrations in the first two days (June 16 to 18) of the sampling period. The high concentration of TN and other–N during the beginning of the sampling period likely coincided with first flush and weekend gardening activities and the washing of hardscapes by homeowners. Flushing of plant debris (containing organic–N) into the drainage network, as well as the runoff of ammonium fertilizer and compost has been reported to be a common contributor of N in urban runoff [42–44]. For remainder of the intensive sampling period (June 19 to June 23), NO_3_–N:TN varied between 0.45 and 1, with the highest fraction occurring each day at around noon. The other–N:TN during the intensive sampling period (June 16 to 23) remained less than 0.60 with the highest fraction occurring near midnight, which corresponded with irrigation with reclaimed water. Other water quality studies in urban areas have shown variation in predominant N forms that are dependent on N sources within the watershed [7, 45]. One explanation of this diurnal switching of N forms could be the use of reclaimed water on common areas in the residential catchment during nighttime hours. Reclaimed water is known to contain higher concentrations of total N and most notably ammonium and organic forms (which are part of other–N) than potable water [46–48].

The mean concentration of TN (10.9 mg L^–1^) found in this study was 40 to 200% higher as compared to other residential runoff studies conducted in the U.S. (Table 2). A study conducted in North Carolina in a small residential catchment (2.54 ha) found an event mean concentration (EMC) of 6.71 mg L^–1^ TN in 69 storm events over the period of 2.3 years [49]. Although reclaimed water likely resulted in greater TN concentration in our study catchment runoff waters, additional factors such as greater impervious area (56%) and slope (15–30%) in our study as compared to 25% impervious area and 10% slope in the Line et al. [49] study might have contributed to this variability. Yang and Toor [3] in a low-density residential catchment (11 ha) in Tampa Bay, Florida found a mean concentration of TN in 25 stormwater runoff events of 0.96 mg L^–1^. The mean NO_3_–N:TN in the study was 0.35 and other–N:TN was 0.65 in stormwater runoff during the wet season [3]. Similarly, Yang and Toor [26] in six medium- and high-density residential catchments found mean concentrations of TN and TP in street stormwater runoff of 0.42 and 0.43 L^–1^, respectively. Further, other–N (mean other–N:TN = 0.61) and PO_4_–P (mean PO_4_–P:TN = 0.63) were the dominant forms of N and P in their street runoff. The greater TN and TP concentrations observed in this study as compared to Yang and Toor [3, 26] study in Tampa Bay, Florida are due to the use of reclaimed water that contains more N and P. The variability in TN and TP concentrations in runoff waters across different studies is attributed to variations in the size of the catchments, climate, irrigation water source (reclaimed vs potable), fertilization practices, and number and timing of samples collection. For example, compared with our study, other studies that showed much lower N concentrations only collected fewer samples and these studies were conducted in areas with mixed land uses and in much larger watersheds. Water quality studies have shown that the size of the watershed can yield varying results for nutrient losses and that a large watershed may provide more biogeochemical opportunities to capture and retain N, keeping it from leaving the area [50, 51]. The high TN concentrations in our study could be further due to the transport of water in a piped conveyance that result in limited interaction of nutrient rich runoff water with pervious areas, use of reclaimed water in common areas, and the absence of rainfall that can dilute the concentrations.

**Table 2:** Comparison of nitrogen, phosphorus, and total suspended solids concentrations in runoff collected from the outflow pipe draining a southern California residential neighborhood with runoff from data from previous studies.

Land Use		TN	TP	TSS	Sampling	Location	Study Area	Reference
		mg L^–1^						
Single Family Residential	Mean	10.85	1.27	52.18	Automated, direct runoff, dry weather, intensive sampling	Aliso Creek Watershed, CA	28.13 ha, 15–30% slopes, 56% ISA^b^ clayey semi-arid, 1 Week	This Study
Single Family Residential	Mean EMC^c^	6.71 ^a^	0.59	73	Automated, stream flow received, flow-weighted, storm runoff for 69 events	Nuese River Basin, NC	2.54 ha, 25% ISA^b^ 2–10% slopes, sandy loam, 2.3 years	[49]
Mixed Residential	Median EMC^c^	2.14^a^–2.46^a^	0.14–0.32	24–61	Periodic storm sampling, stream flow received	Ballona Creek Watershed, CA	30,180 ha, 3 sites, channelized semi-arid, 4 years	[41]
Mixed Residential	Mean	5.16^a^	0.63	19.5	18 dry weather grab samples	Ballona Creek Watershed, CA	30,180 ha, 3 sites, combined channelized, semi-arid, 4 years	[41]
Low-Density Single Family Residential	Mean	0.96	–	–	Automated, direct runoff, wet weather, intensive sampling for 25 events	Tampa Bay, FL	11 ha, 1 site, subtropical, 4 months	[3]
Medium- and High Density Single Family Residential	Mean	0.42	0.43	–	Automated, direct runoff, wet weather, intensive sampling for 21 events	Tampa Bay, FL	3.6–50 ha, 6 sites, subtropical, 2 months	[26]

^a^TN is the sum of Nitrate–N and TKN

^b^ISA is Impervious Surface Area

^c^EMC is mean concentration for flow weighted storm flows

### Phosphorus concentrations and loading in residential runoff

**Fig 5:**
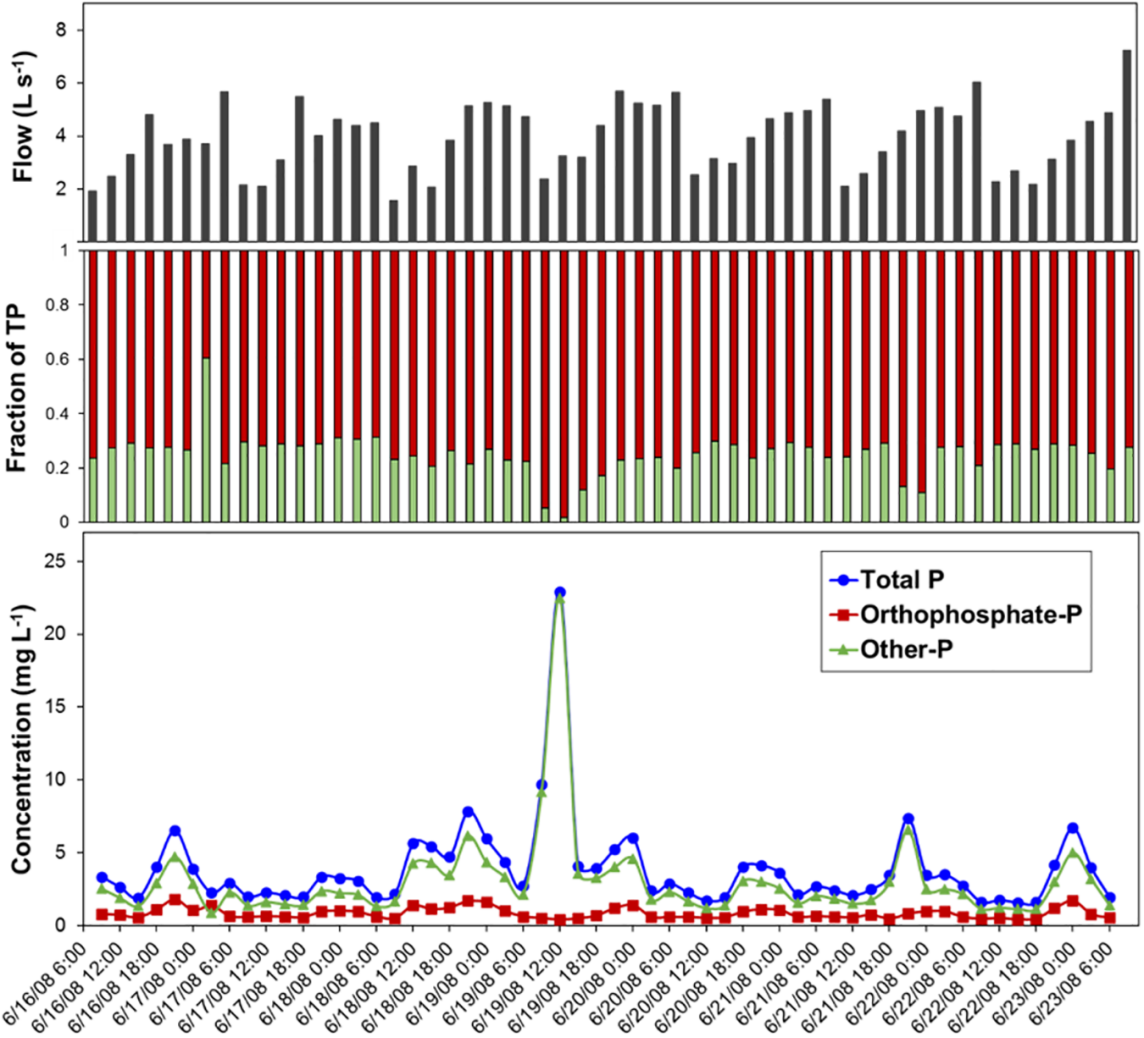
From top to bottom represents measured flow, fraction of PO_4_–P (red) and other–P (green), and concentrations of P forms in residential runoff collected at 3-hour intervals over 56 sampling events from outflow pipe draining a residential catchment during an intensive sampling period of 1-week (June 16 to 23, 2008).

Mean concentrations of TP, PO_4_–P, and other–P (dissolved organic P, particulate P that includes organic and inorganic P) were 1.27, 0.82, and 0.45 mg L^–1^, respectively (Table 1). During the intensive sampling period, mean PO_4_–P:TP was 0.75 and other–P:TP was 0.25. All forms of P reached daily maximum concentrations between 6:00 p.m. to midnight with the exception of one event that occurred around noon on June 19, 2008 where TP was nearly 6-times higher than the recorded mean. This increase in TP was comprised of 95% other–P (Fig 5). This event had little effect on PO_4_–P and indicated that other–P was washed (flushed) into the drainage network. This is probably attributed to the fact that between irrigation events, particulate matter accumulates on the surfaces in the drainage network and when substantial wetting occurs (e.g., storm or hosing off of driveway) particulates are flushed off resulting in marked increase in certain constituent concentrations. The mean TP load was 0.44 kg day^–1^ and was lowest from 6:00 a.m. to noon each day (S3 Table). Similar to P concentrations, TP loads were elevated close to midnight with the exception of one event where TP load was greatest at noon during the middle (June 19, 2008) of the intensive sampling period (Fig 4). The detailed concentrations and proportions data of P forms collected at 3-h intervals over the study period is reported in S5 Table.

Studies have shown that response to “first flush” is greatest for TSS>TP>TN, with particulate and organic forms of P and N being more mobile during this time than dissolved inorganic forms [52–55]. There was also a spike (5-times the mean concentration) in TSS (S2 Fig) 12-h prior to the noted increase in TP, which supports our hypothesis that this increase in TP was related to particulate runoff. It is possible that sediment and organic material (i.e. TSS) transported during the runoff event also transported particulate-P but at a varied time scale influenced by change in flow intensity, suspended particle size, and the speciation of P [25, 56, 57]. Studies show that particulate-P is the dominant form in urban runoff and that accumulation of P in urban regions is mainly a surface issue, whereby particulate-P, associated with the finer fraction (1–25 microns) of sediment, accumulates on impervious surfaces and is then transported into surface waters by rain or irrigation events [58–61]. For example, in our study area, particulate-P and other solids may have accumulated on driveways and sidewalks during the dry season, which can be transported to the drainage network by irrigation. These transient events are significant contributors to nutrient loss from urban environments and can elevate the mean constituent concentrations [62, 63].

In this study, daily P loss predominately consisted of PO_4_–P. For 91% of the 1-wk sampling period, PO_4_–P:TP was 0.50 or more (Fig 5). The reclaimed water used in the residential catchment is known to contain more PO_4_–P than natural or potable water [46, 48, 64–65]. There were two separate occasions (June 19 and June 21, 2008) where other–P was the predominant form. During these two events, P associated with organic matter and the fine fraction of soil sediment was transported with the residential runoff. Although only five runoff samples showed TP to be primarily composed of other–P, this increase was responsible for elevating mean TP concentrations and more than quadrupling P loss during the event. Reclaimed water likely contributed to P loss from our catchment as highest TP concentrations in this residential watershed corresponded to the recommended hours of reclaimed water use (6:00 p.m. to 9:00 a.m.). However, during the sampling period, the transient event (increase in other–P and TP concentration) captured with intensive sampling exerted a greater influence on mean TP concentration than reclaimed water usage.

Mean TP concentration from the catchment was high when compared to other residential water quality studies (Table 2). For comparison, McPherson et al. [41] and Yang and Toor [26] in stormwater runoff found mean TP concentrations of 0.63 and 0.43 mg L^–1^, respectively, which were ~50% of the concentration (1.27 mg L^–1^) found in this study. Yang and Toor [26] reported that PO_4_–P (PO_4_–P: TP = 0.63) was the dominant form in street runoff and suggested that PO_4_–P likely originated from erosion of soil particles and mineralization of organic materials. Total P concentration at the outflow pipe was 7-times greater than the dry weather concentration of TP (0.17 mg L^–1^) found downstream (3 km) from the residential site [31]. Further, mean TP was 13-times greater than the proposed total maximum daily load of 0.1 mg L^–1^ for the Aliso Creek mainstem and several tributaries [66]. This suggests that this and other residential catchments in the watershed are a significant source of P in surface waters.

### Total suspended solids concentrations and loading in residential runoff

Mean TSS during the sampling period was 52.2 mg L^–1^, which increased midway (June 18, 2008) to ~275 mg L^–1^ (Table 1 and S2 Fig) and corresponded to an increase in other–P (and TP, Fig 5) because particulate-P is positively correlated with turbidity and TSS [57, 67]. For the rest of the sampling period, TSS concentrations were below 100 mg L^–1^. The highest concentrations of TSS often occurred from 8:00 p.m. to 6:00 a.m., which corresponded with reclaimed water irrigation times as well as increased flows resulting from runoff generated by residential irrigation systems.

Reclaimed water used in southern California has been shown to contain increased levels of TSS. The lowest concentrations of TSS mirrored reduced flows in the catchment with lowest values occurring at noon each day (S2 Fig). Irrigation driven runoff and transient anthropogenic events (e.g., car washing, erosion, construction, and washing of impervious surfaces) are the likely sources of TSS in this residential catchment. Sediment loss is a function of soil properties, land management, and water characteristics, and the steep and sustained slopes in this residential catchment are conducive to erosion, if not properly managed. Because TSS transport is related to the intensity and duration of runoff events, a decrease in TSS concentration (<20 mg L^–1^) occurred when irrigation ceased each day (near noon) and flow dropped to <2 L s^–1^. Our dry weather TSS concentrations were similar to those studies conducted in wet weather or storm sampling (Table 2). For example, a small (2.54 ha) residential catchment in North Carolina had an EMC TSS of 73 mg L^–1^ (n = 69).

The mean loading rate for TSS was 19.7 kg day^–1^ and the lowest values occurred near noon each day and the highest loads occurred at times that coincided with the recommended reclaimed water irrigation hours i.e. 6:00 p.m. to 6:00 a.m. (S2 Table). However, most HOAs in California irrigate common areas between 11:00 pm and 4:00 am to avoid exposure of reclaimed water to residents that might be using those areas. Like TP, a significant increase in TSS load (nearly 6-times the mean rate) occurred in the middle (June 18 2008) of the sampling period (Fig 4). As mentioned previously, transient events created a flushing effect that is likely responsible for the increase in TSS load that occurred during June 18 to June 19 2008. TSS is closely related to turbidity and this change in water quality during the middle of the intensive sampling period was confirmed by turbidity measurements (data not shown). In summary, nutrient and sediment loading in this catchment was related to anthropogenic activities and reclaimed water use in the catchment. With a few exceptions, all constituents showed greatest losses during the recommended irrigation hours with reclaimed water.

The calculated dry season (153 days) nutrient export rates were 20.2 kg TN ha^–1^, 2.34 kg TP ha^–1^, and 97.1 kg TSS ha^–1^ (S6 Table). Even though export rates from our study area only account for 42% of the year, dry season nutrient export rates calculated for TN and TP were higher than most annual nutrient export rates found in comparable urban runoff studies (S6 Table). Seasonal loads from our study were 2-fold (for TN) and 1.5-fold (for TP) than any literature annual rates for developed lands [68]. In a small residential watershed (2.54 ha) in North Carolina, Line et al. [49] found annual export rates of 23.9, 2.3, and 387 kg ha^–1^ yr^–1^ for TN, TP, and TSS, respectively. It is important to note that California has a Mediterranean climate with dry summers, where most of the rainfall occurs in the period beginning in December and ending in March. Very little, if any, rainfall occurs between June and September. Conservatively, if we assume that wet weather flows from our catchment contribute as much nutrient and sediment as dry weather flows, TN and TP export from the site would still be double the rates found in North Carolina and TSS export would be about half of the export rates. In summary, dry weather flows can make large contributions to annual TN and TP loads particularly in highly irrigated residential catchments. While the dry season load of TSS is less substantial, TSS export from this residential catchment could still impact downstream waters.

### Contribution of reclaimed water use to residential runoff

Reclaimed water is increasingly becoming an important water source in the water scarce southwest. In an effort to conserve high quality potable water, the use of reclaimed water as an alternative source for landscape and agricultural irrigation has become vital to states like California and Florida. Proper management of reclaimed water for irrigation is crucial as reclaimed water is inherently higher in nutrients, suspended solids, dissolved organic matter, and soluble salts [48, 69, 70]. As reclaimed water quality parameters vary by treatment type as well as effluent source, a range of constituent concentrations found in select treatment facilities in California are presented along with potable water quality parameters (S7 Table). The mean concentrations of N and P in our runoff waters were elevated as a result of reclaimed water use within the residential catchment. Mean TN and NO_3_–N are comparable to the concentrations exhibited by reclaimed water. During the intensive sampling period, reclaimed water use had the greatest effect on TN in this residential catchment. While TP and PO_4_–P in runoff were lower than usually found in reclaimed water, TP loss was heavily influenced by transient events (possible construction or landscaping activities) captured with intensive sampling as well as by reclaimed water use. Another indicator that reclaimed water influenced nutrient losses from this neighborhood was the high salinity (soluble salts) and elevated TOC concentrations found during hours of reclaimed water use (S2 Fig). For example, EC measured at the outflow pipe ranged from 1.43 to 3.64 dS m^–1^, which is equivalent to about 915–2330 mg L^–1^ of total dissolved solids. Mean concentration of TOC was 13.1 mg L^–1^, which is typical for wastewater that has received secondary treatment [48]. Frequently, the lowest EC and TOC values occurred at 6:00 a.m., which corresponds to the daily increase in potable water irrigation (S2 Fig).

## Conclusions

Intensive sampling (at 3-h intervals) of runoff waters captured temporal changes that are important in correctly understanding N and P concentrations and forms in residential runoff driven by landscape irrigation. Comparison of this data with other runoff studies illustrates the need to conduct studies at a local catchment scale that considers climate, geography, landscape management, and impervious surface areas. Our results showed that nutrient and sediment loading in the residential catchment was related to anthropogenic activities and reclaimed water use in the site. Further, dry weather flows can make large contributions to TN and TP loads particularly in highly irrigated catchments. Simply classifying land as urban/developed does not provide useful information to accurately predict potential water quality problems. We suggest that dry season or base flow contributions should be evaluated using an intensive sampling approach in urban catchments. For example, if samples were collected using daily grab samples during normal work hours (8:00 a.m. to 4:00 p.m.), mean nutrient export would be underestimated. It is important to consider setting and/or human influences in designing sampling to capture the dynamics of nutrients transport. In setting such as this where a predictable irrigation schedule influences flow and nutrients, a short period of 24-h sampling may be sufficient. However, in other settings the approach may be different depending on temporal/spatial dimension of potential influences. Much of the previous work done to quantify nutrient exports has focused on storm loads as the “first flush” phenomenon has been deemed the primary mechanism of nutrient losses. This may be less true for regions such as semi-arid areas where most of the flows occur in dry conditions in response to landscape irrigation.

## Supporting information

S1 Fig Concentration distributions of nitrogen and phosphorus in residential runoff (n = 56) collected at 3-hour intervals from outflow pipe draining a residential catchment during an intensive sampling period of 1-week (June 16 to 23, 2008). The line represents daily mean concentration and the different letters indicate significant difference (p<0.05).
(TIF)

S2 Fig Concentrations of total suspended solids and total organic carbon, and electrical conductively in residential runoff collected from the outflow pipe draining a residential catchment during an intensive sampling period of 1-week (June 16 to 23, 2008). (TIF)

S1 Table Mean recorded flow at the outflow pipe draining a southern California residential neighborhood during 2007–2008 wet and dry seasons. (PDF)

S2 Table Runoff flow, pH, electrical conductivity (EC), total organic carbon (TOC), and total suspended solids (TSS) concentrations in individual runoff samples collected at 3-hour intervals in June 2008. (PDF)

S3 Table Loads of nitrogen, phosphorus and total suspended solids in runoff collected at the outflow pipe draining a southern California residential neighborhood during one-week of intensive sampling in June 2008. (PDF)

S4 Table Concentrations and proportions of nitrogen forms in individual runoff samples collected at 3-hour intervals in June 2008. (PDF)

S5 Table Concentrations and proportions of phosphorus forms in individual runoff samples collected at 3-hour intervals in June 2008. (PDF)

S6 Table Comparisons of estimated nutrient and sediment export rates from this study and other residential runoff studies. (PDF)

S7 Table Comparison of water quality parameters in residential runoff from this study, treated wastewater (reclaimed water), and potable water from various sources. (PDF)

S1 File Materials and methods. (PDF)

S2 File References. (PDF)
