# Supplementary figures and images for: Managing urban runoff in residential neighborhoods: Nitrogen and phosphorus in lawn irrigation driven runoff
Source: PLoS One. 2017 Jun 12;12(6):e0179151. doi: 10.1371/journal.pone.0179151 (PMC5467952; doi:10.1371/journal.pone.0179151)

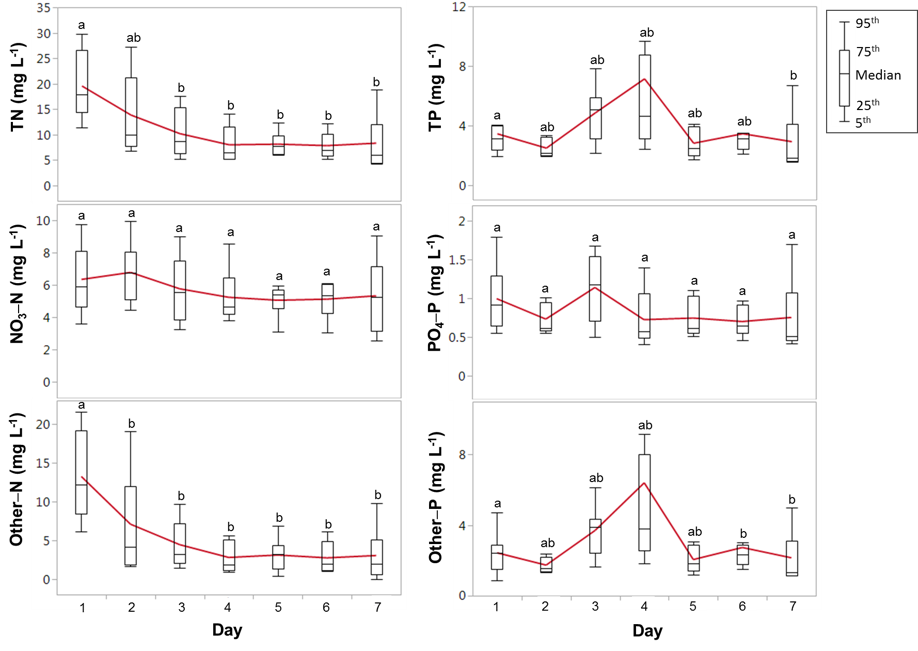

Supplement: S1 Fig — The line represents daily mean concentration and the different letters indicate significant difference (p<0.05). (TIF) [file pone.0179151.s001.tif]

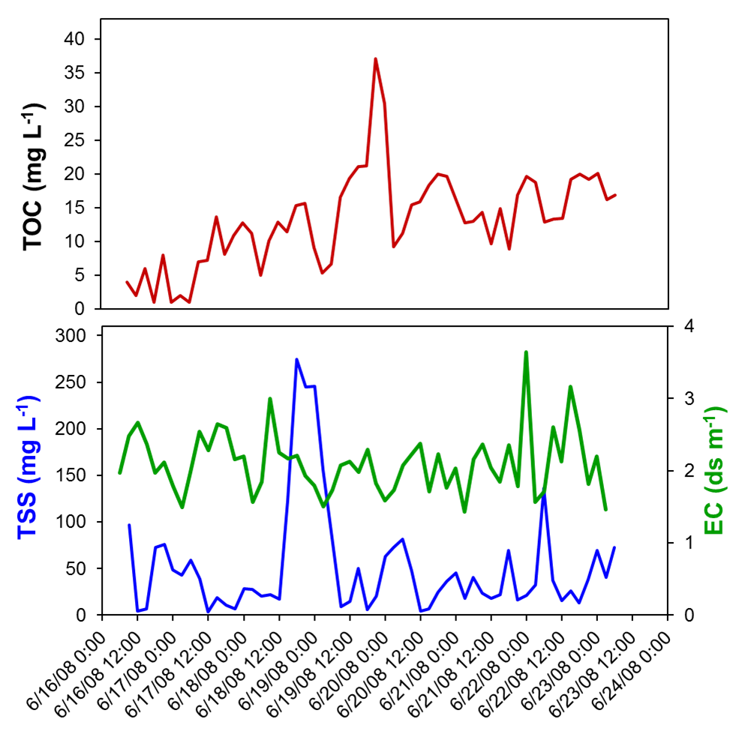

Supplement: S2 Fig — (TIF) [file pone.0179151.s002.tif]
