# Supplementary material for: Managing urban runoff in residential neighborhoods: Nitrogen and phosphorus in lawn irrigation driven runoff
Source: PLoS One. 2017 Jun 12;12(6):e0179151. doi: 10.1371/journal.pone.0179151 (PMC5467952; doi:10.1371/journal.pone.0179151)
Supplement: S1 Table — (PDF) [file pone.0179151.s003.pdf]

S1 Table. Mean recorded flow at the outflow pipe draining a southern California residential neighborhood during 2007–2008 wet and dry seasons.

Season	Month	Flow (L s^{-1})		Total Flow (L)
		Mean	Range	
**Wet**	October 2007	1.93	0.02–49.45	516,131.20
	November 2007	3.27	0.19–151.24	847,558.42
	December 2007	2.65	0.01–99.97	710,055.36
	January 2008	8.18	0.01–364.57	2,189,688.62
	February 2008	4.15	0.01–168.39	1,003,051.30
	March 2008	3.09	0.28–15.84	826,480.81
	April 2008	3.64	0.38–12.60	942,319.14
	Wet season average	3.84	0.01–364.57	7,035,284.86<br>(63% of total)
**Dry**	May 2008	3.53	0.08–29.80	945,297.56
	June 2008	3.30	0.08–9.07	856,231.07
	July 2008	3.75	0.15–17.46	100,4177.65
	August 2008	2.92	0.14–8.61	781,871.26
	September 2008	1.99	0.00–12.20	515,319.36
	Dry season average	3.10	0.00–29.80	4,102,896.90<br>(37% of total)
	Week of intensive sampling	3.96	1.55–7.23	-
