# Supplementary material for: Managing urban runoff in residential neighborhoods: Nitrogen and phosphorus in lawn irrigation driven runoff
Source: PLoS One. 2017 Jun 12;12(6):e0179151. doi: 10.1371/journal.pone.0179151 (PMC5467952; doi:10.1371/journal.pone.0179151)
Supplement: S2 Table — (PDF) [file pone.0179151.s004.pdf]

S2 Table. Runoff flow, pH, electrical conductivity (EC), total organic carbon (TOC), and total suspended solids (TSS) concentrations in individual runoff samples collected at 3-hour intervals in June 2008.

Date/Time	Day	Flow	pH	EC	TOC	TSS
		L s^{-1}		dS m^{-1}	mg L^{-1}	
6/16/08 9:00	1	1.92	7.43	1.97	4.00	96.48
6/16/08 12:00	1	2.48	8.10	2.48	2.00	3.92
6/16/08 15:00	1	3.29	8.13	2.67	6.00	7.08
6/16/08 18:00	1	4.81	8.07	2.36	1.00	72.92
6/16/08 21:00	1	3.68	7.96	1.97	8.00	75.92
6/17/08 0:00	1	3.89	8.02	2.11	1.00	48.60
6/17/08 3:00	1	3.71	8.15	1.79	2.00	43.08
6/17/08 6:00	1	5.66	8.16	1.49	1.00	59.40
6/17/08 9:00	2	2.16	7.86	1.98	7.00	39.24
6/17/08 12:00	2	2.09	7.80	2.54	7.16	3.44
6/17/08 15:00	2	3.11	7.90	2.28	13.60	18.40
6/17/08 18:00	2	5.49	7.85	2.65	8.10	10.76
6/17/08 21:00	2	4.01	7.82	2.59	10.80	6.80
6/18/08 0:00	2	4.64	7.83	2.16	12.70	28.24
6/18/08 3:00	2	4.40	7.86	2.20	11.20	27.84
6/18/08 6:00	2	4.51	8.03	1.57	5.00	20.40
6/18/08 9:00	3	1.55	7.73	1.85	10.10	21.92
6/18/08 12:00	3	2.85	8.03	3.00	12.90	17.28
6/18/08 15:00	3	2.08	8.04	2.25	11.40	123.00
6/18/08 18:00	3	3.84	7.97	2.17	15.30	274.36
6/18/08 21:00	3	5.13	7.78	2.21	15.60	244.48
6/19/08 0:00	3	5.27	7.88	1.93	9.10	245.96
6/19/08 3:00	3	5.13	7.97	1.79	5.30	156.16
6/19/08 6:00	3	4.72	8.07	1.50	6.60	83.28
6/19/08 9:00	4	2.39	8.29	1.74	16.50	9.00
6/19/08 12:00	4	3.26	7.82	2.07	19.30	15.00
6/19/08 15:00	4	3.19	7.90	2.12	21.10	50.20
6/19/08 18:00	4	4.41	8.37	1.98	21.20	5.72
6/19/08 21:00	4	5.70	8.30	2.29	37.10	20.72
6/20/08 0:00	4	5.24	8.32	1.83	30.40	63.36
6/20/08 3:00	4	5.16	8.45	1.59	9.20	72.52
6/20/08 6:00	4	5.64	8.43	1.73	11.20	81.32
6/20/08 9:00	5	2.54	8.57	2.08	15.40	47.88
6/20/08 12:00	5	3.15	8.39	2.22	15.80	4.72
6/20/08 15:00	5	2.97	8.28	2.37	18.30	6.56
6/20/08 18:00	5	3.95	8.14	1.72	20.00	24.16
6/20/08 21:00	5	4.66	7.94	2.23	19.60	36.52
6/21/08 0:00	5	4.89	8.18	1.77	16.20	45.32

6/21/08 3:00	5	4.95	8.40	2.03	12.70	18.12
6/21/08 6:00	5	5.38	8.54	1.43	13.00	40.56
6/21/08 9:00	6	2.11	7.97	2.16	14.30	23.40
6/21/08 12:00	6	2.59	8.26	2.36	9.60	18.28
6/21/08 15:00	6	3.40	8.26	2.05	14.80	22.32
6/21/08 18:00	6	4.19	8.33	1.84	8.80	69.08
6/21/08 21:00	6	4.97	8.33	2.35	16.90	16.00
6/22/08 0:00	6	5.08	8.16	1.78	19.60	21.60
6/22/08 3:00	6	4.76	8.31	3.64	18.70	32.52
6/22/08 6:00	6	6.03	8.54	1.57	12.90	136.32
6/22/08 9:00	7	2.28	8.30	1.71	13.30	36.92
6/22/08 12:00	7	2.70	8.40	2.60	13.40	15.56
6/22/08 15:00	7	2.17	8.51	2.13	19.20	25.64
6/22/08 18:00	7	3.12	8.53	3.16	20.00	13.12
6/22/08 21:00	7	3.82	8.32	2.56	19.20	38.48
6/23/08 0:00	7	4.56	8.28	1.82	20.10	69.28
6/23/08 3:00	7	4.89	8.46	2.19	16.20	40.16
6/23/08 6:00	7	7.23	8.61	1.47	16.90	73.00
