# Supplementary material for: Managing urban runoff in residential neighborhoods: Nitrogen and phosphorus in lawn irrigation driven runoff
Source: PLoS One. 2017 Jun 12;12(6):e0179151. doi: 10.1371/journal.pone.0179151 (PMC5467952; doi:10.1371/journal.pone.0179151)
Supplement: S3 Table — (PDF) [file pone.0179151.s005.pdf]

S3 Table. Loads of nitrogen, phosphorus and total suspended solids (TSS) in runoff collected at the outflow pipe draining a southern California residential neighborhood during one-week of intensive sampling in June 2008.

**Loading Rates**	**Total N**	**Nitrate–N**	**Other–N**	**Total P**	**Orthophosphate–P**	**Other–P**	**Total suspended solids**
	kg day^{-1}						
Mean	3.84	1.95 (51%)[a]	1.89 (49%)[a]	0.44	0.29 (66%)[a]	0.15 (34%)[a]	19.71
Minimum	0.80	0.44	0.02	0.09	0.07	0.01	0.62
Maximum	11.63	4.20	8.94	2.10	0.75	1.99	11.89
Standard Deviation	2.70	0.91	2.08	0.34	0.17	0.28	25.26
Simple Method[b]	3.72	1.94	1.78	0.43	0.28	0.15	17.85

[a] Data in parenthesis are percent of total N or total P load.

[b] Simple method was used to calculate loads by multiplying the mean constituent concentration (mg L^{-1}) by the mean flow (L s^{-1}) for the one-week sampling period [5-6].
