# Supplementary material for: Managing urban runoff in residential neighborhoods: Nitrogen and phosphorus in lawn irrigation driven runoff
Source: PLoS One. 2017 Jun 12;12(6):e0179151. doi: 10.1371/journal.pone.0179151 (PMC5467952; doi:10.1371/journal.pone.0179151)
Supplement: S4 Table — (PDF) [file pone.0179151.s006.pdf]

S4 Table. Concentrations and proportions of nitrogen forms in individual runoff samples collected at 3-hour intervals in June 2008.

Date/Time	Day	TN	Nitrate–N	Other–N	Nitrate–N	Other–N
		mg L^{-1}			% of TN	
6/16/08 9:00	1	11.40	5.29	6.11	46.40	53.60
6/16/08 12:00	1	16.00	7.91	8.09	49.45	50.55
6/16/08 15:00	1	14.60	4.55	10.05	31.19	68.81
6/16/08 18:00	1	28.00	6.46	21.54	23.08	76.92
6/16/08 21:00	1	29.80	9.75	20.05	32.72	67.28
6/17/08 0:00	1	22.50	8.17	14.34	36.29	63.71
6/17/08 3:00	1	14.30	5.01	9.29	35.06	64.94
6/17/08 6:00	1	19.90	3.59	16.31	18.03	81.97
6/17/08 9:00	2	6.75	4.85	1.90	71.90	28.10
6/17/08 12:00	2	10.00	7.34	2.66	73.37	26.63
6/17/08 15:00	2	7.72	5.82	1.90	75.38	24.62
6/17/08 18:00	2	7.76	6.12	1.64	78.84	21.16
6/17/08 21:00	2	21.70	9.94	11.76	45.79	54.21
6/18/08 0:00	2	27.30	8.26	19.04	30.25	69.75
6/18/08 3:00	2	19.50	7.45	12.05	38.22	61.78
6/18/08 6:00	2	10.10	4.44	5.66	43.95	56.05
6/18/08 9:00	3	5.94	3.24	2.70	54.60	45.40
6/18/08 12:00	3	7.63	6.23	1.40	81.69	18.31
6/18/08 15:00	3	8.55	5.24	3.31	61.33	38.67
6/18/08 18:00	3	8.81	5.68	3.13	64.48	35.52
6/18/08 21:00	3	16.70	9.02	7.68	53.99	46.01
6/19/08 0:00	3	17.60	7.91	9.69	44.95	55.05
6/19/08 3:00	3	11.10	5.41	5.70	48.69	51.31
6/19/08 6:00	3	5.27	3.36	1.91	63.72	36.28
6/19/08 9:00	4	5.18	3.80	1.39	73.26	26.74
6/19/08 12:00	4	6.27	4.44	1.83	70.80	29.20
6/19/08 15:00	4	6.77	4.85	1.92	71.68	28.32
6/19/08 18:00	4	9.20	4.88	4.32	53.00	47.00
6/19/08 21:00	4	14.10	8.53	5.57	60.52	39.48
6/20/08 0:00	4	12.30	6.99	5.31	56.85	43.15
6/20/08 3:00	4	5.16	4.16	1.00	80.68	19.32
6/20/08 6:00	4	5.19	4.23	0.96	81.54	18.46
6/20/08 9:00	5	6.03	4.55	1.48	75.52	24.48
6/20/08 12:00	5	6.99	5.70	1.29	81.60	18.40
6/20/08 15:00	5	8.40	4.60	3.80	54.76	45.24
6/20/08 18:00	5	8.93	5.36	3.57	60.01	39.99
6/20/08 21:00	5	12.30	5.47	6.83	44.50	55.50
6/21/08 0:00	5	10.20	5.70	4.50	55.92	44.08
6/21/08 3:00	5	6.39	5.96	0.43	93.22	6.78

6/21/08 6:00	5	5.97	3.08	2.89	51.62	48.38
6/21/08 9:00	6	6.75	5.36	1.39	79.39	20.61
6/21/08 12:00	6	6.42	5.36	1.06	83.47	16.53
6/21/08 15:00	6	10.50	5.04	5.46	47.97	52.03
6/21/08 18:00	6	5.64	3.98	1.66	70.55	29.45
6/21/08 21:00	6	8.96	6.00	2.96	67.00	33.00
6/22/08 0:00	6	7.14	6.07	1.07	85.04	14.96
6/22/08 3:00	6	12.20	6.12	6.08	50.15	49.85
6/22/08 6:00	6	5.24	3.04	2.20	57.94	42.06
6/22/08 9:00	7	4.32	3.45	0.87	79.86	20.14
6/22/08 12:00	7	6.12	2.53	3.59	41.34	58.66
6/22/08 15:00	7	5.88	5.36	0.52	91.14	8.86
6/22/08 18:00	7	4.97	5.11	0.00	100.00	0.00
6/22/08 21:00	7	12.90	7.31	5.59	56.70	43.30
6/23/08 0:00	7	18.80	9.06	9.74	48.20	51.80
6/23/08 3:00	7	9.50	6.74	2.76	70.94	29.06
6/23/08 6:00	7	4.27	3.06	1.21	71.64	28.36
