# Supplementary material for: Managing urban runoff in residential neighborhoods: Nitrogen and phosphorus in lawn irrigation driven runoff
Source: PLoS One. 2017 Jun 12;12(6):e0179151. doi: 10.1371/journal.pone.0179151 (PMC5467952; doi:10.1371/journal.pone.0179151)
Supplement: S5 Table — (PDF) [file pone.0179151.s007.pdf]

S5 Table. Concentrations and proportions of phosphorus forms in individual runoff samples collected at 3-hour intervals in June 2008.

Date/Time	Day	Total P	Orthophosphate –P	Other –P	Orthophosphate –P	Other –P
6/16/08 6:00		mg L^{-1}			% of TP	
6/16/08 9:00	1	3.34	0.79	2.55	23.62	76.38
6/16/08 12:00	1	2.63	0.72	1.91	27.27	72.73
6/16/08 15:00	1	1.90	0.55	1.35	29.00	71.00
6/16/08 18:00	1	4.03	1.10	2.93	27.34	72.66
6/16/08 21:00	1	6.52	1.79	4.73	27.50	72.50
6/17/08 0:00	1	3.89	1.04	2.85	26.73	73.27
6/17/08 3:00	1	2.25	1.36	0.89	60.42	39.58
6/17/08 6:00	1	2.91	0.63	2.28	21.73	78.27
6/17/08 9:00	2	1.96	0.58	1.38	29.61	70.39
6/17/08 12:00	2	2.25	0.63	1.62	28.11	71.89
6/17/08 15:00	2	2.06	0.60	1.46	28.96	71.04
6/17/08 18:00	2	1.96	0.55	1.41	28.11	71.89
6/17/08 21:00	2	3.33	0.96	2.37	28.88	71.12
6/18/08 0:00	2	3.25	1.01	2.24	31.20	68.80
6/18/08 3:00	2	3.06	0.94	2.12	30.68	69.32
6/18/08 6:00	2	1.92	0.60	1.32	31.41	68.59
6/18/08 9:00	3	2.16	0.50	1.66	23.09	76.91
6/18/08 12:00	3	5.64	1.38	4.26	24.39	75.61
6/18/08 15:00	3	5.42	1.12	4.30	20.75	79.25
6/18/08 18:00	3	4.73	1.24	3.49	26.26	73.74
6/18/08 21:00	3	7.84	1.68	6.16	21.46	78.54
6/19/08 0:00	3	5.95	1.60	4.35	26.96	73.04
6/19/08 3:00	3	4.35	1.00	3.35	23.01	76.99
6/19/08 6:00	3	2.73	0.61	2.12	22.33	77.67
6/19/08 9:00	4	9.67	0.51	9.16	5.23	94.77
6/19/08 12:00	4	22.90	0.41	22.49	1.79	98.21
6/19/08 15:00	4	4.05	0.48	3.57	11.91	88.09
6/19/08 18:00	4	3.95	0.67	3.28	17.08	82.92
6/19/08 21:00	4	5.22	1.20	4.02	22.98	77.02
6/20/08 0:00	4	6.00	1.40	4.60	23.31	76.69
6/20/08 3:00	4	2.39	0.57	1.82	24.01	75.99
6/20/08 6:00	4	2.87	0.57	2.30	19.88	80.12
6/20/08 9:00	5	2.24	0.57	1.67	25.61	74.39
6/20/08 12:00	5	1.71	0.51	1.20	29.93	70.07
6/20/08 15:00	5	1.93	0.55	1.38	28.55	71.45
6/20/08 18:00	5	4.02	0.95	3.07	23.60	76.40
6/20/08 21:00	5	4.11	1.11	3.00	27.05	72.95

6/21/08 0:00	5	3.61	1.06	2.55	29.44	70.56
6/21/08 3:00	5	2.13	0.59	1.54	27.55	72.45
6/21/08 6:00	5	2.67	0.64	2.03	23.93	76.07
6/21/08 9:00	6	2.41	0.58	1.83	24.08	75.92
6/21/08 12:00	6	2.07	0.55	1.52	26.77	73.23
6/21/08 15:00	6	2.48	0.72	1.76	29.18	70.82
6/21/08 18:00	6	3.47	0.46	3.01	13.25	86.75
6/21/08 21:00	6	7.38	0.81	6.57	10.91	89.09
6/22/08 0:00	6	3.48	0.96	2.52	27.73	72.27
6/22/08 3:00	6	3.49	0.97	2.52	27.93	72.07
6/22/08 6:00	6	2.74	0.57	2.17	20.94	79.06
6/22/08 9:00	7	1.60	0.46	1.14	28.73	71.27
6/22/08 12:00	7	1.73	0.50	1.23	28.83	71.17
6/22/08 15:00	7	1.57	0.42	1.15	26.99	73.01
6/22/08 18:00	7	1.62	0.47	1.15	28.98	71.02
6/22/08 21:00	7	4.16	1.18	2.98	28.29	71.71
6/23/08 0:00	7	6.70	1.70	5.00	25.40	74.60
6/23/08 3:00	7	3.96	0.78	3.18	19.68	80.32
6/23/08 6:00	7	1.93	0.53	1.40	27.53	72.47
