# Supplementary material for: Managing urban runoff in residential neighborhoods: Nitrogen and phosphorus in lawn irrigation driven runoff
Source: PLoS One. 2017 Jun 12;12(6):e0179151. doi: 10.1371/journal.pone.0179151 (PMC5467952; doi:10.1371/journal.pone.0179151)
Supplement: S6 Table — (PDF) [file pone.0179151.s008.pdf]

S6 Table. Comparisons of estimated nutrient and sediment export rates from this study and other residential runoff studies.

Land Use	TN	TP	TSS	Sampling	Location	Study Area	Reference
	————kg ha^{-1} yr^{-1}————						
Single Family Residential	20.20[a]	2.34[a]	97.13[a]	Automated time sensitive intensive sampling for 1 week during dry weather	Aliso Creek Watershed, CA	28.13 ha<br>15-30% slopes<br>56% ISA[b]<br>clayey<br>semi-arid	This study
Residential	23.9	2.3	387	Automated flow-weighted storm runoff for 69 events	Nuese River Basin, NC	2.54 ha<br>25% ISA[b]<br>2-10% slopes<br>sandy loam<br>humid subtropical	[7]
Residential	6.0	0.4	–	Storm sampling	Charlotte and Mecklenburg County, NC	humid subtropical	[8]
Residential	6.7	0.96	–	Automated storm sampling of 43 events	Chesapeake Bay	62.11 ha (2 sites)<br>18% ISA<br>humid subtropical	[9]
Residential	8.4	1.3	–	Storm Sampling	Nationwide	81 sites in 22 cities	[10]
Developed	5.0–9.72	0.45–1.5	–	Literature Review	-	78 individual studies	[11]

[a] Export rates are in kg ha^{-1} ds^{-1} (ds= dry season of 153 days). [b]ISA = Impervious surface area
