# Supplementary material for: Managing urban runoff in residential neighborhoods: Nitrogen and phosphorus in lawn irrigation driven runoff
Source: PLoS One. 2017 Jun 12;12(6):e0179151. doi: 10.1371/journal.pone.0179151 (PMC5467952; doi:10.1371/journal.pone.0179151)
Supplement: S7 Table — (PDF) [file pone.0179151.s009.pdf]

S7 Table. Comparison of water quality parameters in residential runoff from this study, treated wastewater (reclaimed water), and potable water from various sources.

Water Quality Constituent	Mean residential runoff concentration (This study)	Reclaimed water[b]	Potable water
pH	8.15	6.8–7.6	7.0–8.6[c]
EC (dS m^{-1})	2.11	1.02–1.44	0.27[d]
Total nitrogen (mg L^{-1})	10.85	17.2–24.9	1.4[d]
NH_4–N (mg L^{-1})	–[a]	1.4–25	–
NO_3–N (mg L^{-1})	5.66	0.7–21.3	0–0.4[c]
Organic–N (mg L^{-1})	–[a]	0.2–2.6	–
Total phosphorus (mg L^{-1})	1.26	12.5	0.25[d]
Orthophosphate–P (mg L^{-1})	0.83	3.4–30.8	–
Total dissolved Solids (mg L^{-1})	1350	476–940	440–490[c]
Total Suspended Solids (mg L^{-1})	52.18	1–26	1.0[d]

[a]Mean values of NH_4–N plus organic-N were 5.2 mg L^{-1}.
[b]Mean concentration ranges of secondary and tertiary treated reclaimed water from select facilities in California [12]
[c]Moulton Niguel Water Quality Data [13]
[d] Water used to irrigate turfgrass plots in Virginia [14]
