# Supplementary material for: Managing urban runoff in residential neighborhoods: Nitrogen and phosphorus in lawn irrigation driven runoff
Source: PLoS One. 2017 Jun 12;12(6):e0179151. doi: 10.1371/journal.pone.0179151 (PMC5467952; doi:10.1371/journal.pone.0179151)
Supplement: S1 File — (PDF) [file pone.0179151.s010.pdf]

Materials and methods

Site description

The main waterway in the watershed is Aliso Creek, which drains a long, narrow coastal canyon with headwaters in the Cleveland National Forest and ultimately discharge to the Pacific Ocean at Aliso Beach. The watershed includes portions of the cities of Aliso Viejo, Laguna Beach, Laguna Hills, Laguna Niguel, Laguna Woods, Lake Forest, and Mission Viejo [1]. The watershed covers approximately 7,844 ha with roughly 45.5% of the land (3,572 ha) classified as residential, with remainder land uses as public (2,385 ha, 30.4%), and commercial (964 ha, 12.3%) [2]. The watershed is located in the San Joaquin Hills, in the coastal northwestern portion of California's Peninsular Ranges geomorphic province. The San Joaquin Hills consist primarily of Miocene and Pliocene age marine sedimentary rocks that have been uplifted, faulted, and dissected by stream erosion [3].

Flow measurement

It was determined that positive measured flows at the outflow pipe were usable when compared to calculated flows using the following Manning formula:

$$Q = \frac{KAR^{2/3}S^{1/2}}{n}$$

where, Q is the flow rate (L s^{-1}), K is a constant dependent on units, A is the cross sectional area of the pipe (m^2), R is the hydraulic radius (m) (an expression of A p^{-1}, or cross sectional area of water, A, divided by the wetted perimeter, *p*), S is pipe slope (m m^{-1}), and *n* is a roughness coefficient corresponding to the texture of the pipe [4]. Constant low flows were observed at the outflow pipe during the intensive sampling. Onsite observations using a float

indicated that runoff water from an irrigation event could reach the outflow pipe in less than 30-min. No rainfall events occurred during the intensive sampling period of 1-wk (June 16 to 23 2008).
