# Supplementary material for: Managing urban runoff in residential neighborhoods: Nitrogen and phosphorus in lawn irrigation driven runoff
Source: PLoS One. 2017 Jun 12;12(6):e0179151. doi: 10.1371/journal.pone.0179151 (PMC5467952; doi:10.1371/journal.pone.0179151)
Supplement: S2 File — (PDF) [file pone.0179151.s011.pdf]

Supplementary Information References

1. County, O. Proposed model for 2007 Aliso Creek watershed action plan; 2006.

2. Pappas P. Aliso creek watershed land use. Orange County Public Works, Orange County Public Works; 2009.

3. Consulting ME. Final report of geotechnical evaluation for environmental impact report. proposed Aliso Creek Golf Course and Resort. Laguna Beach, California; 2007.

4. Manning R. On the flow of waters in open channels and pipes. Transactions of Civil Engineers of Ireland 1891; 20 :161-207.

5. Endreny TA, Hassett JM, Wolosoff SE. Robustness of pollutant loading estimators for sample size reduction in a suburban watershed. International Journal of River Basin Management 2005; 3 :53-66.

6. Li H, Lee JHW, Cai M. Nutrient load estimation methods for rivers. International Journal of Sediment Research 2003; 18 :346-351.

7. Line DE, White NM, Osmond DL, Jennings GD, Mojonnier CB. Pollutant export from various land uses in the upper Neuse River basin. Water Environment Research 2002; 74 :100-108.

8. Bales JD, Weaver JC, Robinson JB. Relation of land use to stream flow and water quality at selected sites in the city of Charlotte and Mecklenburg County, North Carolina; 1999.

9. Hartigan JP, Quasenbarth TF, Southerland E. Calibration of NPS model loading factors. Journal of Environmental Engineering 1983. 109

10. U.S. EPA. Results from the nationwide urban runoff program, *In* W. P. Division, (ed.), Vol. 1, Final Report ed. U.S. Environmental Protection Agency, Washington, D.C; 1983.

11. Dodd RC, McMahon G, Stichter S. Watershed planning in the Albemarle-Pamlico estuarine system: average annual nutrient budgets. Research Triangle Institute, Research Triangle Park, NC; 1992.

12. Asano T, Tchobanglous G. Municipal wastewater treatment and Eeffluent utilization for irrigation. FAO, Rome; 1987.

13. District MNW. Drinking water quality report. 2012.

14. Evanylo G, Ervin E, Zhang X. Reclaimed water for turfgrass irrigation. Water 2010; 2 :685-701.
